# In Situ Disentangling Local Thermal and Photoelectron Contribution in Photoelectrochemical Reactions Based on Optical Microfiber Sensors

**DOI:** 10.1002/advs.202521585

**Published:** 2025-12-12

**Authors:** Guangzheng Luo, Benfang Xu, Tiansheng Huang, Li‐Peng Sun, Bai‐Ou Guan

**Affiliations:** ^1^ Institute of Photonics Technology Guangdong Provincial Key Laboratory of Optical Fiber Sensing and Communications Jinan University Guangzhou 510632 China; ^2^ College of Physics & Optoelectronic Engineering Jinan University Guangzhou 510632 China; ^3^ School of Physics and Optoelectronics Xiangtan University Xiangtan 411105 China

**Keywords:** in situ decoupling, optical microfiber sensor, photoelectrochemical, photoelectronic effect, photothermal effect

## Abstract

The introduction of the photothermal effect holds significant importance for enhancing the efficiency of solar‐driven photoelectrochemical energy conversion, including water splitting, CO_2_ reduction, and nitrogen reduction reactions. Distinguishing and quantifying the effects of local heating to photoelectrochemical reactions, however, remains challenging. Herein, it is reported that the local thermal in photoelectrochemical reaction can be in situ measured via an optical microfiber sensor, which captures thermo‐optic signals from highly sensitive modal interference. For proof‐of‐concept, the laser‐induced graphene (LIG) electrode as a model for photoelectrochemical reaction, which has emerged as a highly promising photoelectrode material owing its excellent photothermal conversion efficiency, tunable structure, and outstanding electrical conductivity. Experimental studies demonstrate that the local photothermal effects contribution can be quantified in real time, which further decoupling the photoelectronic effect contribution in photocurrent. Additionally, the potential for further enhancing sensitivity through dispersion turning point is demonstrated, achieving approximately five times the improvement over traditional fiber interferometers. This advancement holds promise for ultra‐sensitive detection of weak photoelectrochemical reactions. Therefore, this work provides critical experimental evidence for decoupling the photothermal and photoelectronic effects in photoelectrochemical reactions through a highly sensitive microfiber in situ detection technique, facilitating the development of advanced photoelectric materials and more efficient solar energy conversion systems.

## Graphical abstract

An in situ optical microfiber sensor capable of real‐time decoupling of photothermal and photoelectronic effects in photoelectrochemical reactions is presented. The approach utilizes highly sensitive modal interference to dynamically capture localized thermo‐optic signals from photothermal materials, thereby enabling quantitative discrimination of photothermal contributions and extraction of photoelectronic responses. This technique provides a critical tool for mechanistic investigation and performance optimization in photoelectrochemical energy conversion systems.

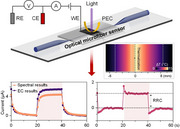

## 1 Introduction

The intensifying global challenges of energy scarcity and environmental pollution underscore the critical imperative for developing alternative clean energy sources and green, sustainable environmental remediation technologies.^[^ ^1^ ^]^ Solar energy, as a clean and renewable resource, has attracted much attention due to its potential for efficient and sustainable energy conversion via photoelectrochemical processes.^[^ ^2^, ^3^, ^4^, ^5^, ^6^ ^]^ In conventional photoelectrochemical systems, solar radiation is absorbed by semiconductors, generating electron‐hole pairs that drive chemical reactions; however, this process is frequently constrained by several interconnected factors. The inherently sluggish surface reaction kinetics not only limits the overall catalytic efficiency but also promotes undesirable side reactions, such as photocorrosion, which degrades the photoelectrode.^[^ ^7^ ^]^ Furthermore, bulk and interfacial defects within the semiconductor act as recombination centers, leading to high electron‐hole recombination rates that severely reduce the quantum efficiency.^[^ ^8^ ^]^ These problems and other optical losses together lead to limited solar energy utilization, which in turn leads to unsatisfactory photoelectrochemical energy conversion efficiency.^[^ ^9^ ^]^ Consequently, various strategies such as defect engineering,^[^ ^10^ ^]^ doping,^[^ ^11^ ^]^ heterojunction construction,^[^ ^12^ ^]^ and the introduction of magnetic^[^ ^1^, ^13^ ^]^ and thermal fields^[^ ^14^, ^15^ ^]^ have been explored to enhance photoelectrochemical performance. Among these, introducing the photothermal effect into photoelectrochemical systems is undoubtedly the most promising approaches. The photothermal effect exploits photothermal materials (e.g., noble metals, semiconductors, carbon‐based materials, organic compounds) to absorb photons and efficiently convert them into thermal energy.^[^ ^16^, ^17^, ^18^ ^]^ This localized heat at active sites effectively lowers reaction energy barriers and facilitates charge transfer, which can significantly enhance the catalytic performance through a synergistic photothermal effect.^[^ ^19^ ^]^ However, it is noteworthy that the role of thermal effects is characterized by a dual nature: in most instances, localized heating significantly enhances the reaction performance. For example, a thermo‐electrochemical investigation of a Si‐based photoelectrochemical flow cell system within the 25–65 °C range demonstrated that the observed increase in current density and the reduction in photovoltage originate from the temperature variation.^[^ ^20^ ^]^ Conversely, under specific systems or conditions, thermal effects can induce detrimental consequences; for instance, photovoltaic cells experience a voltage loss of ≈100 mV during peak hours due to thermal recombination, thereby leading to an insufficient driving force for redox reactions.^[^ ^21^ ^]^ Consequently, the precise measurement and regulation of thermal effects in photoelectrochemical energy systems are of paramount importance.

The synergistic interaction between photothermal and photoelectric effects improves the overall efficiency of photoelectrochemical reactions. Generally speaking, the reaction efficiency can be directly evaluated by the current intensity, but traditional current measurement method only reflects the combined contribution of these effects. This makes it difficult to discriminate between or decouple the relative contributions of local thermal and photoelectronic effects for photoelectrochemical reaction efficiency. Meanwhile, since the reaction occurs at the electrode materials interface inside the electrochemical cell, conventional ex‐situ methods such as thermal imaging and thermocouples are unable to accurately capture the rapid temperature changes at the material interface.^[^ ^22^, ^23^ ^]^ Thus, disentangling these two effects remains a considerable challenge. Although a significant amount of research has focused on this issue in recent years, the precise mechanisms for quantifying thermal and photoelectronic effects remain subject to considerable debate and divergence.^[^ ^24^ ^]^ Ex‐situ measurements are susceptible to external environmental interference, leading to deviations in recorded information and may consequently result in misjudgments (exaggerations or neglect) of the contributions of these two effects.^[^ ^25^ ^]^ Therefore, to accurately measure the thermal effect that generate on the catalyst surface and decouple the photoelectronic effect regarding the photoelectrochemical processes, developing an in situ, time‐resolved, highly sensitive temperature detection technology are needed.

Optical fiber sensors, owing to their exceptional physicochemical merits such as high flexibility, miniature dimensions, real‐time detection capability, and remarkable sensitivity,^[^ ^26^, ^27^ ^]^ provide an advanced platform for in situ monitoring techniques that enable the precise decoding of chemical/electrochemical reaction events. Moreover, designs based on various structures such as fiber Bragg gratings,^[^ ^28^, ^29^ ^]^ fiber‐optic surface plasmon resonance,^[^ ^30^, ^31^ ^]^ and optical microfiber interferometer^[^ ^32^ ^]^ have achieved breakthrough progress in in situ analysis, making microfiber sensing extremely promising in quantifying localized thermal effects in photoelectrochemical reactions.

Herein, we proposed an in situ monitoring approach for decoupling photothermal effect during photoelectrochemical reactions utilizing an optical fiber microfiber Mach‐Zehnder interferometer (MZI), which capture thermo‐optic signals from highly sensitive modal interface. A photoelectrochemical reaction system was constructed by using laser‐induced graphene (LIG) electrode as photothermal material. By correlating high temporal resolution photocurrent measurements with simultaneously acquired temperature data through calibration and fitting, we successfully quantified the contribution of thermal effects to the total photocurrent. In addition, the photoelectronic contribution to the LIG photocurrent was independently decoupled. The results suggest that the developed MZI device has excellent in situ measurement ability and reliability compare to conventional ex situ methods, providing a more precise strategy of actual surface temperature variations measurement during operation. Additionally, by fabricating a dispersion turning point microfiber with a smaller diameter, the temperature sensitivity is significantly enhanced, resulting in a substantial increase in the wavelength shift from 10 to 50 nm within a temperature variation of 2 °C, compared to conventional MZI devices. This work demonstrates a way to quantitatively study thermal effects by accurately measuring the actual reaction temperature, guiding the performance evaluation and mechanistic understanding of the photothermal contributions in photoelectrochemical systems.

## 2 Results and Discussion

### 2.1 Photoelectrochemical Studies of LIG

**Figure 1:**
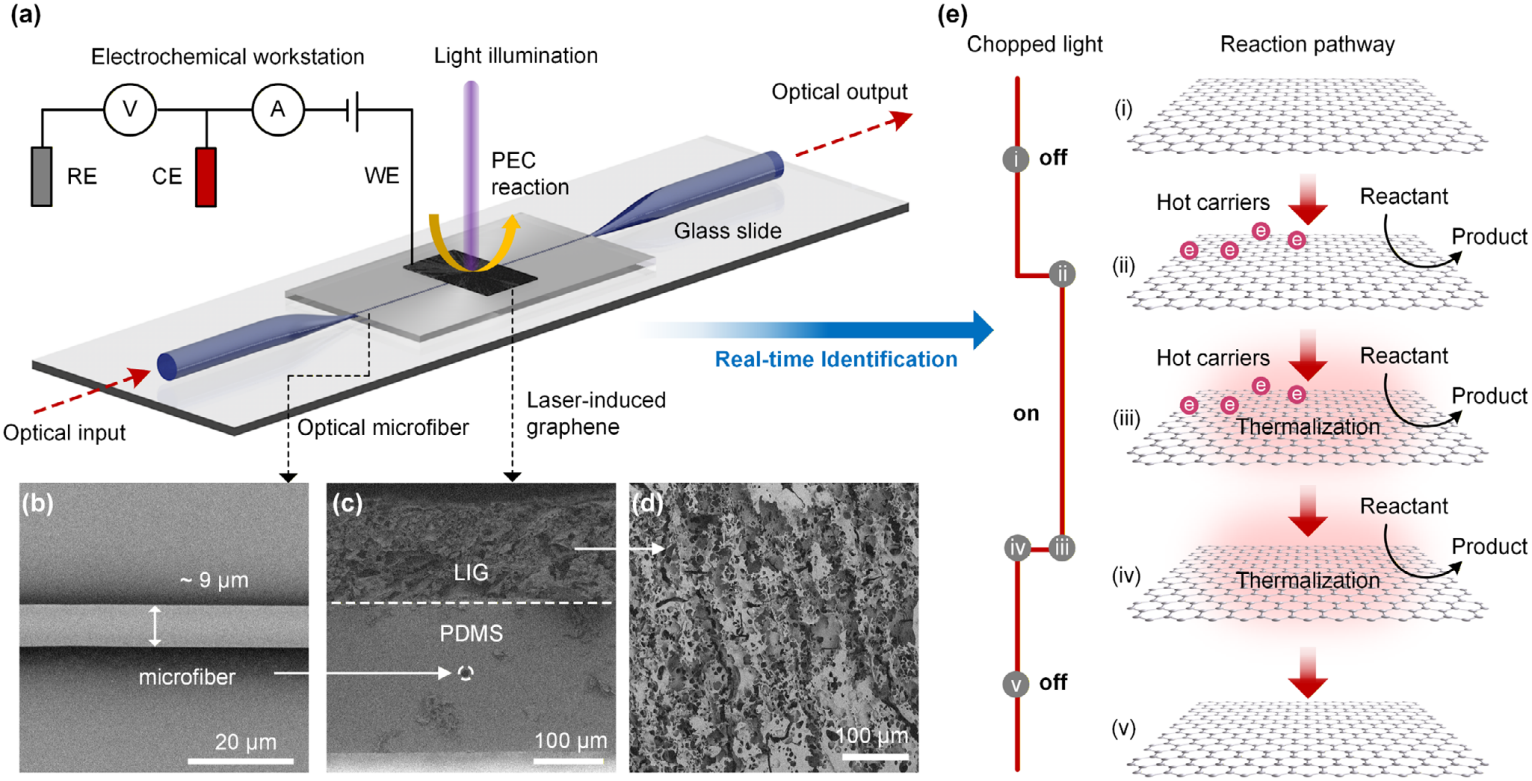
a) Schematic diagram of the experimental device structure. SEM images of b) the uniform region of the microfiber MZI, c) Longitudinal cut of PDMS‐MZI‐LIG composite structure, and d) PI‐LIG. e) The process of photoelectric effect produced by the chopped light irradiation of LIG.

**Figure** **1**a illustrates the proposed fiber‐optic sensing system for operando investigates of photoelectrochemical reaction processes. As shown in Figure 1b, a microfiber (diameter ∼9 µm) with tapered ends and a uniform waist was fabricated in this work, and the sensitivity the prepared devices is verified by using standard refractive index liquids (Figure S1a,b, see Supporting Information for details). Furthermore, to eliminate the influence of laser irradiation on the photoelectrochemical reaction system, the proposed MZI interferometer was continuously irradiated with the maximum laser power of 405 nm laser for 120 min, verifying its good stability. The standard deviation of wavelength shift during the entire irradiation process was only 72 pm (Figure S2, Supporting Information). To achieve in situ measurement of the thermal effects in photoelectrochemical reactions, the MZI devices were encapsulated by polydimethylsiloxane (PDMS) (Figure S3, Supporting Information), which serves as an ideal temperature sensitive material owing to the pronounced thermo‐optic properties.^[^ ^33^, ^34^ ^]^ The temperature sensing specificity of the PDMS‐encapsulated microfiber was systematically evaluated. As illustrated in Figure S4a (Supporting Information), the device exhibits a pronounced blue shift in the interference spectrum with increasing temperature, demonstrating a high sensitivity of 3.49 nm °C^−1^ and excellent linearity (R^2^ = 0.9914) within the range of 26.5–60 °C (Figure S4b, Supporting Information red line). This behavior is attributed to the thermo‐optic effect and thermal expansion of the PDMS matrix. Furthermore, environmental refractive index testing of the encapsulation structure revealed that PMDS effectively isolates the sensor from interference caused by changes in the environmental refractive index, further confirming its temperature selectivity (Figure S4b, Supporting Information purple line). These results validate that the PDMS encapsulation strategy not only preserves high thermal sensitivity but also eliminates interference from external refractive index changes, rendering the sensor highly suitable for accurate temperature monitoring in complex electrochemical environments. A cross‐sectional SEM image of the PDMS‐MZI‐LIG assembled structure is presented in Figure 1c. From bottom to top, the microfiber encapsulated within PDMS and the LIG layer derived from laser‐processed PI. Notably, the microfiber is positioned near the center of the PDMS layer and remains in close proximity (∼100 µm) to the uppermost LIG interface, thereby enabling real‐time, in situ monitoring of temperature variations during the LIG thermal effect. As shown in Figure 1d, the LIG film exhibits a classic porous nanosheet‐like morphology, consistent with results of previously works.^[^ ^35^ ^]^ LIG's porous structure significantly increases the specific surface area, providing abundant active sites for electrochemical reactions. Furthermore, Figure S5 (Supporting Information) presents the Raman spectra of the as‐prepared LIG, confirming the successful graphene formation via laser‐induced graphitization. Figure S6 (Supporting Information) shows the electrochemical performance of the as‐prepared LIG, demonstrating that the as‐prepared LIG electrode possesses superior charge transfer capabilities, providing a foundation for subsequent in situ photoelectrochemical reaction study.

Figure 1e presents the photoelectrochemical processes induced by chopped light, wherein the high‐energy electron‐hole pairs generated in the LIG electrode under photoexcitation undergo rapid recombination on picosecond timescales, with these charge carriers directly participating in and influencing electrochemical reactions.^[^ ^36^, ^37^ ^]^ This is consistent with the commonly observed phenomenon in semiconductor electrodes or plasma metal nanostructures, where photocurrents generated by energetic charge carriers (electrons and holes) respond immediately under illumination and usually peak in millisecond time (denoted as ii in Figure 1e). In contrast, the thermal effect‐induced heating of the local environment requires a considerably longer duration to reach thermal diffusion equilibrium (iii). Upon cessation of illumination, the photoelectronic effect disappears rapidly, whereas the elevated temperature decays more gradually (iv).^[^ ^38^ ^]^

**Figure 2:**
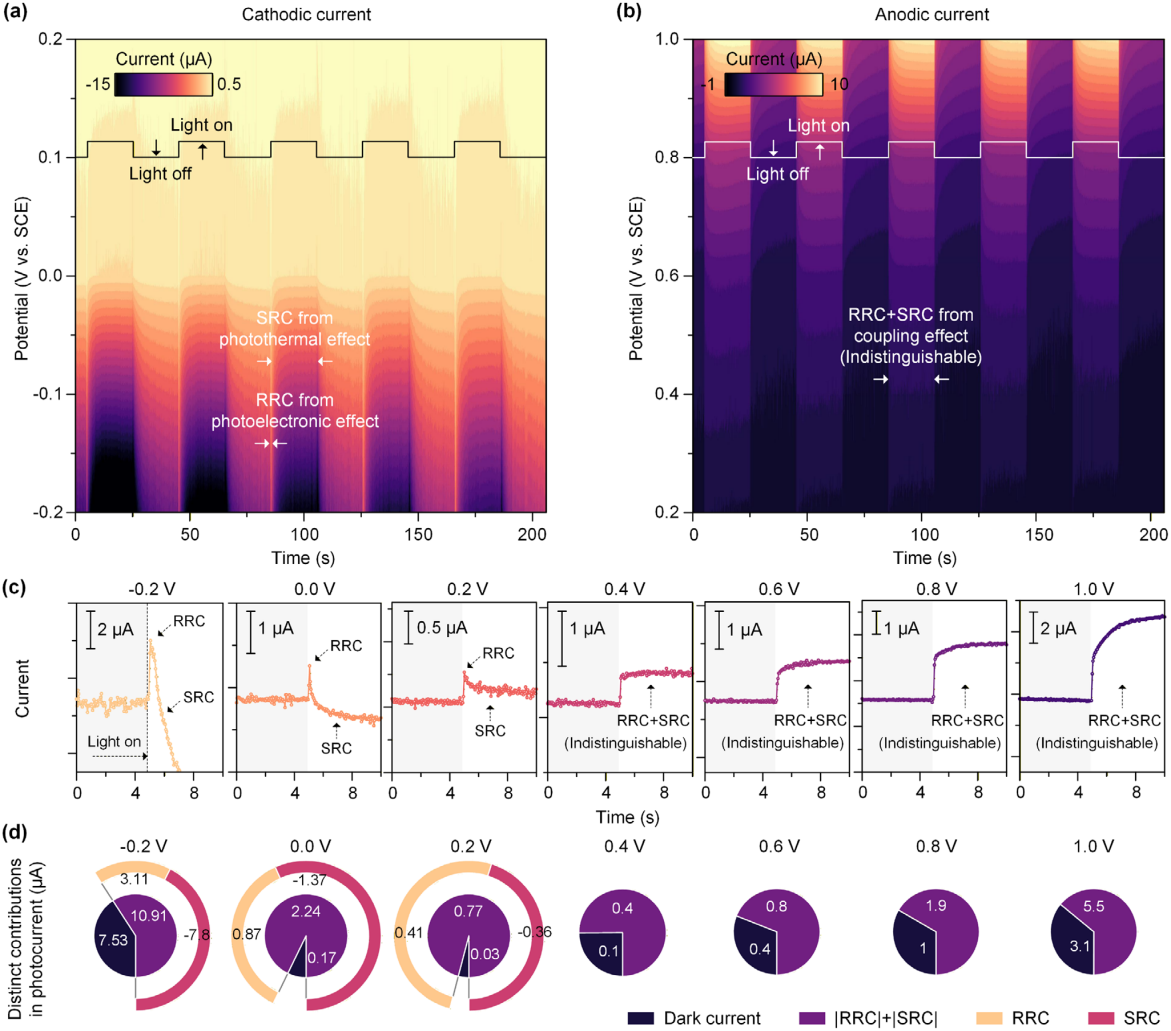
Photocurrent‐time curves of the LIG electrode at applied negative a) and positive b) potentials. c) Zoomed‐in images of the LIG photocurrent within 10 s before and after laser light on at different bias voltages. d) The components of the LIG photocurrent generation process include dark current, RRC, and SRC.

The photoelectrochemical behavior of LIG electrode was systematically investigated by using a laser source with a wavelength of 405 nm (power density of 586 mW cm^−2^) and a 20 s on‐off cycle. As illustrated in **Figure** **2**a,b, the transient photocurrent response (i–t curve) of the LIG electrode under laser illumination was examined over a range of bias voltages from −0.2 to 1.0 V. Under laser irradiation, the photocurrent intensity generated at negative potential (cathode) and positive potential (anode) increased significantly with the increase of bias voltage. This is attributed to the effect of applied potential on the separation and transport efficiency of photogenerated charge carriers.^[^ ^39^ ^]^ While the quantity of photogenerated carriers remains relatively constant under same laser power, variations in the external bias alter the migration and recombination kinetics, thereby modulating the number of carriers participating in the external circuit current and consequently affecting the overall photocurrent value. Specifically, as the applied voltage increases, the separation efficiency of photogenerated carriers increases, and the recombination efficiency decreases, so that the photocurrent gradually increases.^[^ ^40^ ^]^ A zoomed view of the photocurrent changes during the 10‐second intervals before and after laser switching (Figure 2c) reveals a rapid increase upon illumination followed by a gradual decay before stabilization. The photocurrent curve can be composed of two parts: a rapid response current (RRC) operating on a timescale of ≈0.05 s, and a slow response current (SRC) evolving over ≈10 s. These components correspond respectively to the photoelectric and thermal effects contributing to the current in the external circuit.^[^ ^38^, ^41^ ^]^ Accordingly, the existence of RRC and SRC is distinctly observable at biases of −0.2, 0.0, and 0.2 V. However, as the voltage increases to 0.4–1.0 V, the two parts of the current become increasingly inseparable. The proportional contributions of these current components under various bias voltages are summarized in Figure 2d. In this system, the dark current primarily stems from Faradaic processes, including the redox reactions of oxygen‐containing functional groups on the LIG surface and the reduction of dissolved oxygen in the electrolyte (Figure S7, Supporting Information). Given the widely recognized chemical stability of LIG, the contribution from corrosion current is considered negligible. Notably, to eliminate any potential influence of the MZI sensor itself on the photocurrent measurements, a control experiment was performed by comparing the photocurrent responses of the same LIG electrode with and without the integrated MZI device within more than 200 s. As shown in Figure S8 (Supporting Information), the photocurrent intensity of the LIG electrode with and without the MZI device hardly changed, indicating that the integration of the MZI on the LIG electrode does not interfere with the photocurrent measurement. Similarly, while the contributions of RRC and SRC can be readily discriminated at lower biases, at higher voltages only the relationship between the background current and the total photocurrent can be ascertained, rendering the decoupling of photothermal and photoelectric effects unfeasible. Therefore, this study proposes an in situ real‐time monitoring of LIG electrode surface temperature variations through microfibers, thereby enabling precise quantification of the photothermal contribution to the photocurrent.

### 2.2 In Situ Microfiber Detection of Photothermal Effect of LIG Electrode

**Figure 3:**
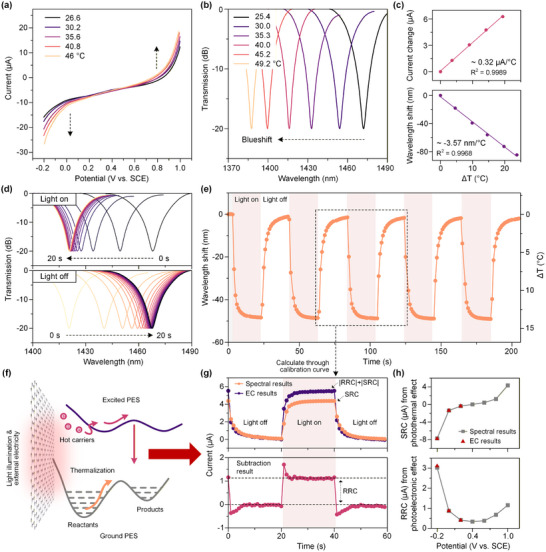
a) The I‐V curves of LIG at different temperatures, scanning rate of 0.05 V s^−1^. b) The change of interference peak position of MZI device under different temperature conditions. c) The temperature dependence of the LIG current and the wavelength shift of the MZI device obtained from (a) and (b), respectively. d) The wavelength variation of MZI‐LIG under laser on–off. e) Spectral drift and temperature variation under laser periodic on‐off cycles in 200 s. f) Schematic of the photoelectronic response and photothermal effect chemical reaction pathway of LIG. g) SRC and RRC results calculated from the calibration curve. h) Comparison of SRC and RRC results for electrochemical and optical measurements at bias voltages of ‐0.2– 1.0 V.

To establish a quantitative relationship between the spectral drift of the MZI device and the SRC current, temperature‐control calibration of the LIG electrode was initially performed on a heating platform. With a thermometer positioned in close proximity to the surface region of the LIG electrode, a series of temperature gradients were applied for heating, and electrochemical measurements were conducted after the temperature had stabilized. **Figure** **3**a shows that the I‐V curve of the LIG electrode at a bias voltage of 1.0 V varies regularly with increasing temperature (*T*). By fitting the data, a current–temperature relationship was obtained (Equation 1: I  =  a*T*+ b, where a and b are constants), exhibiting a sensitivity of ≈0.32 µA °C^−1^ (Figure 3c, top). Similarly, temperature calibration was carried out for the proposed MZI device, wherein the interference spectra were recorded after thermal equilibrium was achieved at each preset temperature. Throughout this process, spectral data of the MZI device were acquired at different temperatures (Figure 3b). With the increase of temperature, the wavelength position of the interference peak of MZI gradually blue shifted. A linear fitting between the wavelength drift (Δ*λ*) and the temperature change (Δ*T*) yielded Equation 2: Δ*T* =  cΔλ+ d, where c and d are constants, with a sensitivity of ∼ 3.57 nm °C^−1^ and a strong linear correlation (Figure 3c, bottom).

Subsequently, the MZI device was placed in a quartz electrolytic cell containing a 0.2 m Na_2_SO_4_ solution, and the LIG surface was irradiated under a 40 s laser switching cycle to trigger the photothermal effect. The interference spectral information was recorded in real time, and the results are presented in Figure 3d: a rapid blue shift in the interference peak wavelength was observed within the first 20 s after laser is turned on, followed by a gradual stabilization, indicating that the temperature variation induced by the photothermal effect during the photoelectrochemical process reached equilibrium; conversely, a swift red shift occurred within 20 s after laser is turned off, with the wavelength gradually returning to its initial position. The wavelength shift was converted into temperature variation based on Equation 2 (Figure 3e), revealing that during the 20‐second laser irradiation period, the interference peak wavelength of the MZI blue‐shifted by ≈47 nm, the corresponding temperature increased rapidly and gradually stabilized at a rise of ≈12 °C. Moreover, the wavelength shifts and temperature variations exhibited high synchrony repeated 200 s laser on‐off cycles, demonstrating excellent reproducibility and reliability of the MZI device in monitoring the photothermal effect of LIG.

To investigate the SRC resulting from sustained heating during the photoelectrochemical reaction at the LIG electrode, the obtained temperature change (red curve) was fitted according to Equation 1 to derive the corresponding current variation (black curve), as illustrated in Figure S9 (Supporting Information). Obviously, under a power density of 586 mW cm^−^^2^ and a voltage of 1.0 V, the photothermal effect at the LIG electrode induced an SRC of ≈3 µA. These results clearly reflect the outstanding photothermal conversion performance of LIG, thereby establishing a foundation for in situ quantitative decoupling of photothermal and photoelectronic effect in the photoelectrochemical response of LIG electrodes.

In the photoelectrochemical reaction mediated by carbon‐based photothermal material LIG, the observed photothermal effect exhibits a high dependence on π to π* orbital, owing to its strong light absorption capacity and abundant conjugated π‐bonds. As shown in Figure 3f, when the material is illuminated by light that matches the possible electronic transitions in the molecule, the generated high energy carriers will transition from the ground state to a higher energy level orbital, thereby significantly increasing the reaction rate.^[^ ^42^ ^]^ The relaxation of excited state electrons occurs via an electron–phonon coupling mechanism, whereby the absorbed photon energy is transferred from the excited state electrons to vibrational modes of the entire atomic lattice, resulting in a macroscopic temperature increase of the material.^[^ ^43^, ^44^, ^45^, ^46^ ^]^ This process contributes to a reduction in the activation energy of the reaction and facilitates the separation of photogenerated charges, thereby improving the overall catalytic performance.^[^ ^47^ ^]^ The photocurrent curve obtained from experiment indicates that both the photoelectronic and photothermal effects of LIG can influence the chemical reaction and its response on different time scales, which can be quantitatively distinguished by photoelectrochemical methods. At a bias voltage of 1.0 V, electrochemical measurements alone can only obtain the total photocurrent result (│RRC+SRC│). Based on the temperature data acquired through in‐situ MZI monitoring, the SRC component was calculated by Equation 1, as represented by the yellow curve in Figure 3g. Simultaneously, subtracting the SRC from the overall photocurrent (purple curve) enables quantitative decoupling of the RRC (red curve). Through this method, we have successfully decoupled the photothermal contribution within the photoelectrochemical process of LIG, obtaining current components of RRC and SRC, thereby separating the photoelectronic and photothermal effects. In addition, we decoupled the SRC and RRC results between −0.2 to 1.0 V voltages, and the results obtained between −0.2 and 0.2 V are consistent with those from electrochemical measurements. Compared to conventional electrochemical techniques, the proposed MZI device demonstrates the capability to quantitatively distinguish SRC from RRC even at bias voltages exceeding 0.2 V (Figure 3h).

### 2.3 Dependence of Photocurrent on Incident Light

**Figure 4:**
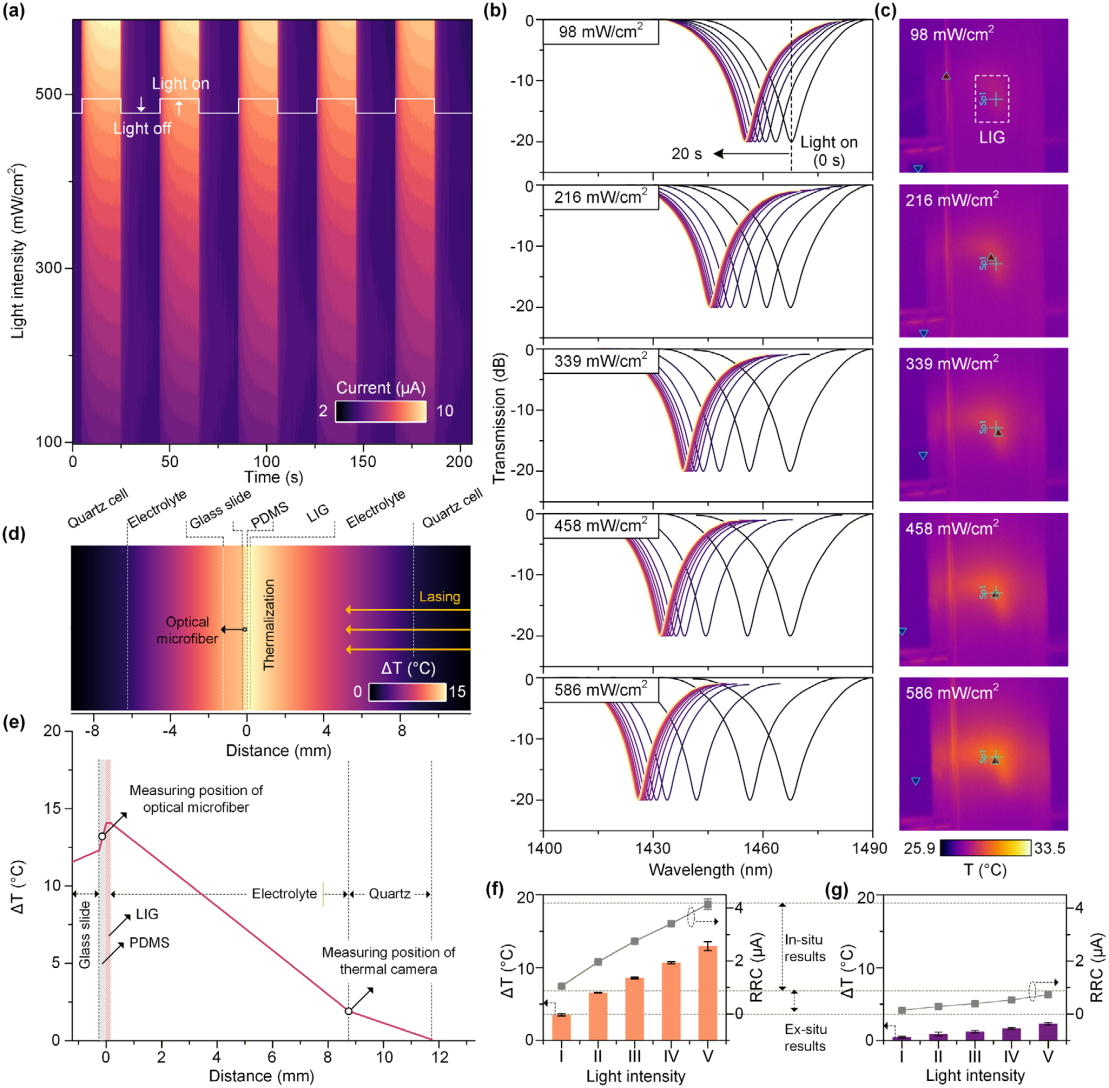
a) The photocurrent response of LIG at the incident light intensity. b) The wavelength shift of the MZI interference peak and c) infrared thermal imager temperature changes for LIG photocurrent measurements (0–20 s) at different laser powers (I: 98 mW cm^−2^; II:216 mW cm^−2^; III:339 mW cm^−2^; IV:458 mW cm^−2^; V:586 mW cm^−2^). d) Simulation results of the thermal distribution at the photoelectrochemical reaction interface based on finite element methods. e) Corresponding simulated temperatures of the LIG electrode at different distances in the photoelectrochemical reaction cell. Comparison of the temperature variations obtained by f) the MZI device and g) infrared thermal imager under different laser powers and the converted RRC results.

To further investigate the dependence of photocurrent on light intensity, chronoamperometric (i–t) measurements were conducted under a bias voltage of 1.0 V. The resulting photocurrent signals generated by LIG are illustrated in **Figure** **4**a, demonstrating a progressive enhancement with increasing laser power. Simultaneously, both the MZI and a conventional infrared thermal imager were employed to record the spectral shift variations (Figure 4b) and the temperature changes on the LIG electrode surface (Figure 4c) over a 20 s period under different power density. The comparison results reveals that the MZI device enables in‐situ, real‐time, and highly sensitive monitoring of temperature variations on the LIG electrode surface, accurately capturing the dynamic process of thermal changes. In contrast, the infrared thermal imager can only ex‐situ measure temperature values as demonstrated in the simulation result (Figure 4d,e), making it difficult to track transient thermal evolution in real time. Thus, the MZI device exhibits significant advantages in monitoring temperature related to photothermal effects.

Further fitting and conversion of data obtained from both methods obtained corresponding temperature values and RRC results (Figure 4f,g). Interestingly, within the laser power density range of 98 to 586 mW cm^−^^2^, the temperature variations and RRC values measured by the MZI were significantly higher than those acquired via infrared thermography. It is further confirmed that MZI has a higher sensitivity to temperature fluctuation for LIG electrode. This capability is attributed to the PDMS‐encapsulated MZI being positioned ≈100 µm directly beneath the LIG thin film, thereby enabling real‐time in‐situ temperature monitoring. Figure 4d,e presents simulated thermal distribution profiles at the reaction interface. Evidently, an increased distance between the temperature measurement position and the LIG electrode leads to greater temperature deviation, particularly when a medium such as a quartz dish is interposed between the temperature sensor and the electrode, resulting in considerable discrepancy between the measured temperature and the actual thermal changes near the LIG surface. Consequently, temperature data obtained through infrared imaging may fail to reflect the true temperature on the LIG surface, which could lead to misinterpretation of the contribution of photothermal effects. In comparison, owing to its in‐situ measurement capability, the MZI allows precise characterization of dynamic temperature evolution at the reaction interface, providing critical technical support for accurate differentiation and decoupling of photothermal contributions.

**Figure 5:**
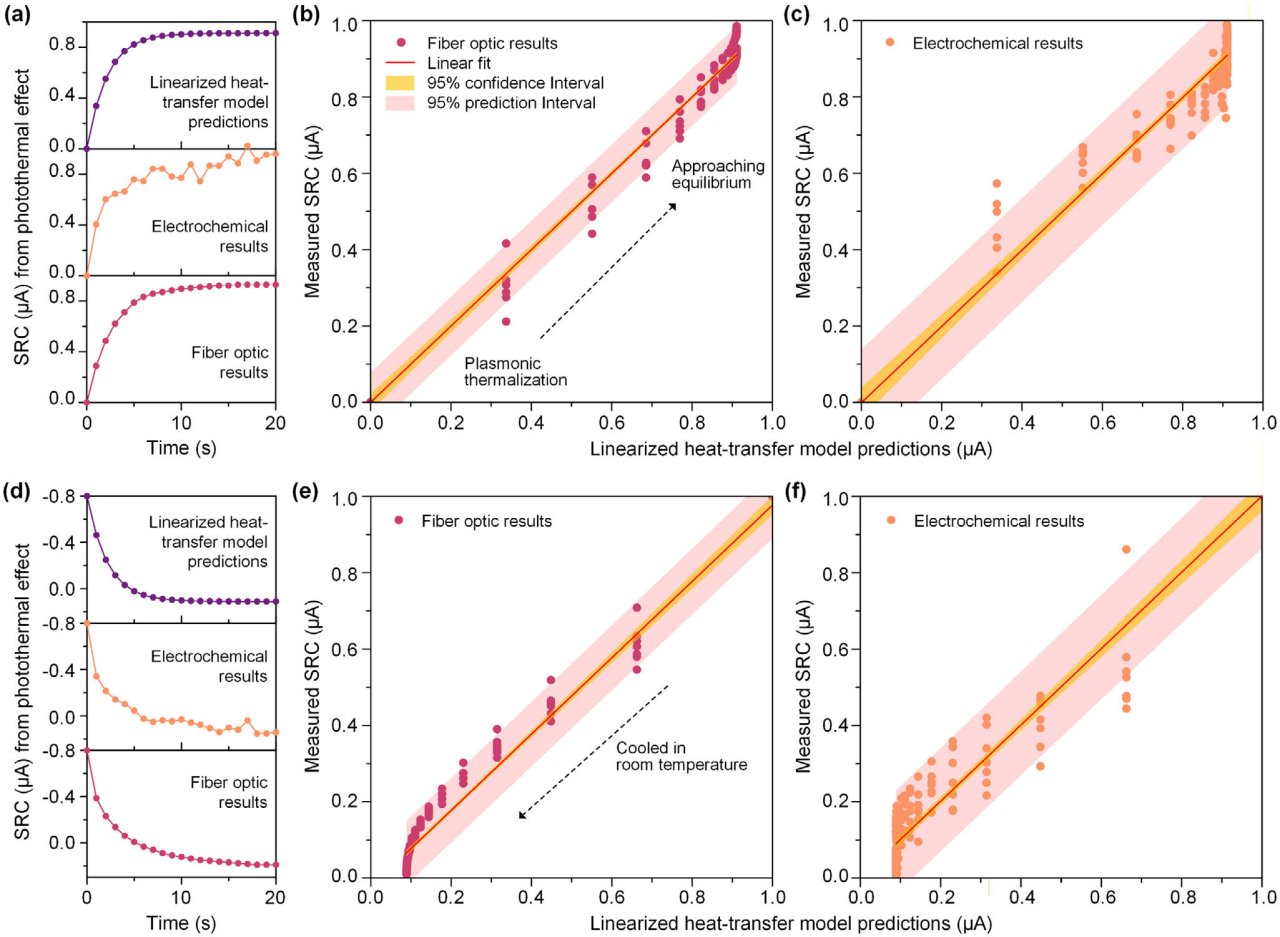
Correlation analysis of the model constructed based on the SRC current measured at 1.0 V using MZI optical and electrochemical methods. a) Fitting functions for model predictions (purple), electrochemical measurements (orange), and optical measurements (red) under illumination (0–20 s); Degree of fit between the true and predicted values of b) the fiber optic results and c) electrochemical result under illumination; d) Fitting functions for model predictions (purple), electrochemical measurements (orange), and optical measurements (red) in the dark; Degree of fit between the true and predicted values of e) the fiber optic results and f) electrochemical results in the dark. The error bars represent the standard deviation.

Moreover, we validated that the SRC current originates from the photothermal effects of LIG electrodes by constructing a thermal conversion predictive model. Postulating that the SRC results from the temperature increase associated with the photothermal effects of the LIG electrode, which follows a temperature–time profile, the corresponding expression can be derived within a thermodynamic framework based on linear non‐equilibrium thermodynamics for an open system heated under constant light intensity:^[^ ^38^ ^]^ $T=Ae^{-\frac{\mathrm{akt}}{\mathrm{Cl}}}+\frac{Pl}{\mathrm{ak}}+T_{0}$, where *A*, a, k, *C*, l、*P* and *T*_0_ represent a constant determined by boundary conditions, electrode dimensions, thermal conductivity, heat capacity, thickness of the thermal diffusion layer, incident light power, and ambient temperature (regarded as constant), respectively. Assuming *T* = *T_0_* at t = 0, Equation 3 is obtained: $A\;=-\frac{Pl}{\mathrm{ak}}$, where *A* must be negative since the incident light power input *P* is positive. Conversely, when the incident light energy is negative (under dark conditions), *A* is positive. As shown in Figure 5a, d, fiber optical results are in more significant agreement with the model prediction results than electrochemical results, indicating superior accuracy of the optically determined SRC. **Figure** **5**b,e illustrates the correlation between the SRC measured by the proposed optical strategy and the model‐predicted values under light and dark conditions, respectively, with both confidence and prediction intervals narrowly distributed around the fitted curves, indicating strong linear relationships. In contrast, the SRC values obtained via electrochemical measurements exhibit marked deviations from the model predictions under both illuminated (Figure 5c) and dark conditions (Figure 5f), accompanied by a discernible non‐zero intercept, which underscores the inherent limitations of conventional electrochemical approaches in decoupling photothermal contributions. These differences likely originate from dark current backgrounds or other slow electrochemical processes that exhibit weak temperature dependence and are not fully captured by the model. As the photocurrent measured in electrochemical systems represents an aggregated signal from multiple concurrent processes, it remains challenging to accurately isolate the SRC component attributable solely to the photothermal effect. These findings collectively demonstrate that the proposed optical method enables more precise quantification of the photothermal contribution, unaffected by complex electrochemical background signals, thereby offering superior accuracy and reliability compared to fitting based solely on electrochemical data. In addition, the experiment briefly demonstrates the proposed MZI interferometer for monitoring the photothermal effect of Ag and Ag/CuO electrodes during the photoelectrochemical reaction process (Figures S10 and S11, see Supporting Information for details), which further proves the applicability of this technique in situ analysis of the photothermal effect of different materials during the photoelectrochemical reaction process.

### 2.4 Dispersion Turning Point‐Enhanced Microfiber Detection of Photothermal Effect

**Figure 6:**
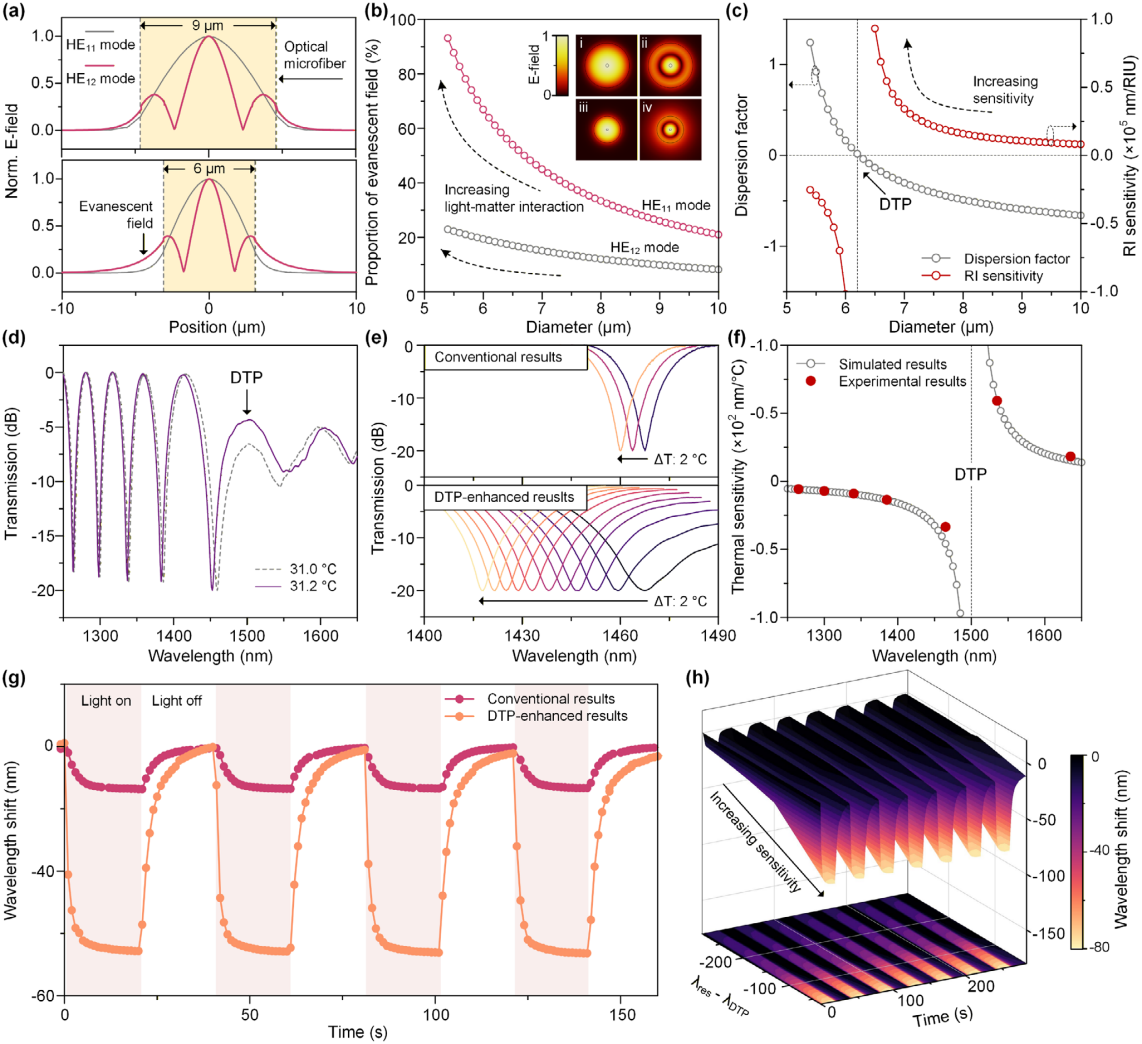
a) Calculated mode field distributions for the HE_11_ and HE_12_ modes at 9 µm (top) and 6 µm (bottom) diameters (These field intensity results are normalized). b) Calculated evanescent field contributions to different HE_11_ and HE_12_ modes for microfiber waveguides of different diameters (Insets: the calculated field intensity distributions of the HE_11_ and HE_12_ modes at 9 µm diameter (i‐ii) and 6 µm diameter (iii‐iv)). c) The calculated dispersion factor and RI sensitivity as a function of the fiber diameter. Under a 2 °C temperature change, d) spectral response of the proposed DTP‐enhanced microfiber and e) comparison of spectral shifts of the MZI device and the DTP‐enhanced microfiber; f) Comparison of theoretically calculated and experimentally acquired temperature sensitivities; g) Comparison of spectral shifts of a conventional MZI and a DTP‐sensitized micro‐nanofiber in photoelectrochemical reactions under low power; h) Relationship between the resonant wavelength of a conventional MZI and the DTP wavelength spacing and the response sensitivity.

As indicated by formula (1) in the experimental section, the refractive index sensitivity S of the microfiber interferometer is mainly determined by variations in *λ*, *Γ*, Δn, and *n_ext_*, with the latter three parameters being modulated by the microfiber diameter. Therefore, we fabricated a microfiber with a reduced diameter of 6 µm by fused taper, aiming to enhance the temperature monitoring sensitivity. According to the numerical simulations, the 1D electric field distributions of the conventional microfiber sensor (9 µm diameter) and the proposed 6 µm diameter sensor are obtained (**Figure** **6**a), where the yellow shaded area represents the waveguide region, while the blank area corresponds to the ambient medium. Figure 6b simulated the evanescent field ratio for microfiber waveguides of different diameters. The results demonstrated a sharp increase in the evanescent field ratio as the diameter decreases and approaches the wavelength scale. Under such conditions, the binding effect of the original waveguide core on the optical field is weakened, resulting in the formation of a novel waveguide structure, together constituted by the cladding and the surrounding medium, which significantly enhances the light‐matter interaction and thereby improves the sensitivity. Furthermore, when the fiber diameter reaches a specific size, the group effective refractive index difference of the interference modes approaches zero. At this point, the refractive index sensitivity exhibits a sharp increase resembling an antiresonance pattern (Figure 6c). Since it is related to waveguide dispersion, this enhancement behavior is also called the dispersion turning point (DTP).^[^ ^32^ ^]^ The above calculation results confirm that, compared to traditional microfiber sensors, the proposed DTP‐enhanced microfiber sensor exhibits more significant optical field distribution and superior refractive index sensitivity in the ambient medium.

Leveraging this high refractive index sensitivity, the DTP‐enhanced microfibers with the same PDMS encapsulation and LIG preparation processes are anticipated to further improve the performance of in‐situ photothermal effect monitoring. As depicted in Figure 6d, the DTP‐enhanced microfiber can induce significant spectral shift within a narrow temperature range of 0.2 °C. Figure 6e compares the temperature response characteristics of the two types of devices: in the 2 °C temperature range, the conventional MZI device exhibits a spectral shift of less than 10 nm, whereas the DTP microfiber demonstrated a larger shift of almost 50 nm, indicating a remarkable enhancement in sensitivity. The theoretically simulated temperature sensitivity of the DTP microfiber in Figure 6f was in excellent agreement with the experimental data (solid dots). To validate the advantage of the DTP‐enhanced microfiber for temperature measurement during the LIG photoelectrochemical process, this study compared the wavelength response to temperature changes of the two devices at a low laser power intensity (98 mW cm^−2^) (Figure 6g). The results indicated that the wavelength shift of the conventional MZI device is only ≈15 nm, which may lead to measurement inaccuracies of the photothermal effect at this or lower power. In contrast, the DTP‐enhanced microfiber achieves a wavelength shift of ≈65 nm, significantly outperforming conventional MZI device. Figure 6h further reveals that the response sensitivity increases as the MZI resonance wavelength approaches the DTP wavelength position (*λ_res_* *λ_DTP_*). These results fully demonstrated that the DTP‐enhanced microfiber possesses higher temperature sensitivity at low‐power laser irradiation, enabling more precise decoupling of the photothermal effect in LIG‐based photoelectrochemical reactions.

## 3 Conclusion

In summary, this work proposed an optical microfiber MZI than can capture the photothermal signal generated by highly sensitive modal interface for in situ, real‐time monitoring of localized temperature during photoelectrochemical processes, which enabled the discrimination and quantification of the contribution of localized thermal effects and photoelectronic effects to the photocurrent. By integrating the optical microfiber sensor with a LIG electrode, a sensing platform capable of simultaneous electrochemical testing and optical temperature measurement was constructed, significantly enhancing the detection accuracy of the real temperature at the reaction interface. The experimental results demonstrated that the MZI device can accurately capture the dynamic temperature change of LIG during photoelectrochemical reactions. Through calibration of the temperature–current relationship, the spectral shift was converted into the slow‐response current induced by the photothermal effect, thereby successfully decoupling and quantifying the relative contributions of photothermal and photoelectronic effects to the overall photocurrent. Notably, under higher bias conditions, this strategy remains effective in decoupling the two effects, outperforming the resolution of traditional electrochemical approaches. To further enhance the detection sensitivity for minute temperature variations, a DTP‐enhanced microfiber with a diameter of 6 µm was designed and fabricated. By optimizing the mode field distribution and increasing the evanescent field proportion, this structure achieves approximately ∼5‐fold improvement in temperature sensitivity, significantly boosting the monitoring capability and decoupling accuracy for photothermal effects under low‐power illumination. This study established a novel pathway for the precise quantification of photothermal contributions in photoelectrochemical processes and provides valuable insights for the rational design of photothermal systems applicable in solar‐driven photoelectrochemical energy conversion.

## 4 Experimental Section

### Microfiber Preparation

In this work, an MZI was prepared by the flame–brushing tapering technique, and the whole operation process was carried out on a high‐precision displacement platform (M–403, Physik Instruments) controlled by a controller (C–863, Physik Instruments). Firstly, a section of the coating‐stripped single‐mode fiber (SMF‐28, Corning) was thoroughly cleaned with anhydrous ethanol and kept in a dry condition. Then, the cleaned fiber was fixed to the optical fiber fixture of micro‐displacement stages at both ends, ensuring that the stripped region was positioned centrally between the two fixtures. One end of the fiber was connected to a broadband light source (BBS, 1250–1650 nm, Golight), while the other end was coupled to an optical spectrum analyzer (OSA, AQ6370D, Yokogawa), enabling real‐time monitoring of the spectral characteristics of the fiber device during the preparation process. During the fiber tapering process, a stabilized butane flame was applied to heat the central region of the stripped fiber at an appropriate distance. After several seconds of high temperature heating, the fiber gradually softened and reached a molten state. Subsequently, the electric displacement platform was immediately activated to move the two platforms in opposite directions along the horizontal axis, thereby stretching the fiber and ultimately yielding a microfiber with a uniform waist diameter of ∼ 9 µm.

### Principle of Microfiber

To achieve highly sensitive thermal effect measurements, an optical microfiber with a biconical structure was designed by the fused biconical taper. This biconical configuration effectively breaks the adiabaticity, thereby forming a classic MZI.^[^ ^32^, ^48^ ^]^ The phase difference *φ* of the interferometer can be expressed as *φ* =  (2π/*λ*)Δ*nL*, where *∆n* = *n_eff1_‐n_eff2_* denotes the effective refractive index difference between the fundamental HE_11_ mode and the higher order HE_12_ mode, and *L* represents the interaction length of the modes. When incident light propagates through the transition and uniform regions of a total length *L* in an optical fiber, a phase difference is generated and accumulated between the HE_11_ and HE_12_ modes. The magnitude of this phase difference is closely related to the geometric structure of the microfiber. By taking the partial derivative of the external refractive index (RI) and combining it with the phase difference *φ*, the sensitivity equation for refractive index sensing can be derived as:^[^ ^49^ ^]^

(1) $$S=\frac{\partial \lambda }{\partial n_{\mathrm{ext}}}=\lambda \frac{1}{\Gamma }(\frac{1}{\Delta n}\frac{\partial \Delta n}{\partial n_{\mathrm{ext}}})$$

where $\Gamma =1-\frac{\lambda }{\Delta n}\frac{\partial \Delta n}{\partial \lambda }$ represents the dispersion factor, which characterizes the effect of the refractive index difference on the wavelength ∂Δ*n*/∂λ. As indicated in formula (1), the refractive index sensitivity *S* of the MZI is collectively determined by the wavelength *λ*, the dispersion factor *Γ*, and external refractive index induced effective refractive index difference ∂Δn/∂n_ext_, with both *Γ* and ∂Δ*n*/∂*n*_ext_ being dependent on the diameter of the microfiber.

## Conflict of Interest

The authors declare no conflict of interest.

## Supporting information



Supporting Information

## Data Availability

The data that support the findings of this study are available from the corresponding author upon reasonable request.
