# Supplementary material for: In Situ Disentangling Local Thermal and Photoelectron Contribution in Photoelectrochemical Reactions Based on Optical Microfiber Sensors
Source: Adv Sci (Weinh). 2025 Dec 12;13(12):e21585. doi: 10.1002/advs.202521585 (PMC12948228; doi:10.1002/advs.202521585)
Supplement: Supplementary file 1 — Supporting Information [file ADVS-13-e21585-s001.docx]

Supporting Information

**In-situ disentangling local thermal and photoelectron contribution in photoelectrochemical reactions based on optical microfibers sensor**

*Guangzheng Luo, Benfang Xu, Tiansheng Huang, Li-Peng Sun^*^, Bai-Ou Guan^*^*

Guangzheng Luo, Benfang Xu, Li-Peng Sun, Bai-Ou Guan

Institute of Photonics Technology, Guangdong Provincial Key Laboratory of Optical Fiber Sensing and Communication, Jinan University, Guangzhou 510632, China

College of Physics & Optoelectronic Engineering, Jinan University, Guangzhou 510632, China

E-mail: lpsun@jnu.edu.cn (L. P. Sun), tguanbo@jnu.edu.cn (B.-O. Guan)

Tiansheng Huang

School of Physics and Optoelectronics

Xiangtan University

Xiangtan 411105, China

Funding: This work was supported by the National Natural Science Foundation of China (62575129, 62175090), the Guangdong Basic and Applied Basic Research Foundation (2024A1515011846), the Local Innovative and Research Teams Project of Guangdong Pearl River Talents Program (2019BT02X105)

**Table of Contents**

[1. Materials and reagents 3](#_Toc215321138)

[2. Characterization techniques 3](#_Toc215321139)

[3. Response of refractive index sensing in microfiber MZI devices 3](#_Toc215321140)

[4. The stability of MZI devices 5](#_Toc215321142)

[5. The process of PDMS encapsulation of microfiber sensors 6](#_Toc215321143)

[6. Performance evaluation of encapsulated microfiber sensors 7](#_Toc215321144)

[7. Preparation of Laser-Induced graphene 9](#_Toc215321145)

[8. Performance Characterization of LIG 9](#_Toc215321146)

[9. Photocurrent comparison between PDMS-MZI device and PDMS-MZI-LIG 13](#_Toc215321147)

[10. Conversion of temperature variation into current variation 13](#_Toc215321148)

[11. In-situ microfiber detection of photothermal effect of Ag and Ag/CuO electrode 14](#_Toc215321149)

# 1. Materials and reagents

PDMS (Sylgard 184, Dow Corning) was purchased from Guangzhou Zock Biotechnology Development Co., Ltd. KCl, Potassium chloride, potassium ferricyanide, potassium ferrocyanide, sodium sulfate anhydrous were all purchased from Macklin Biochemical Co., Ltd. (Shanghai, China). Sucrose was purchased from Aladdin Biochemical Technology Co., Ltd. (Shanghai, China).

# 2. Characterization techniques

The dimensions of the prepared optical fiber and LIG electrode are characterized by a scanning electron microscope (Phenom Pure, Phenom). (Photo)electrochemical testing via electrochemical workstation (CHI 660E, Shanghai Chenhua) control. The prepared LIG was used as the working electrode, the saturated calomel electrode (CHI 150, Shanghai Chenhua) and the platinum sheet (99.99%, Taizhou Zenno Material Technology) were used as the reference electrode and the counter electrode, respectively, and 0.2 M Na_2_SO_4_ was used as the neutral electrolyte. The LIG electrode is characterized by confocal Raman imaging system (DXR, Thermo Scientific). For Raman spectroscopy tests, the samples are focused through an Olympus LMPlanFL 50× microscope objective. The Raman spectrum is then acquired after 10 seconds of laser excitation at a power of 2 mW and a wavelength of 785 nm.

# 3. Response of refractive index sensing in microfiber MZI devices

To evaluate the refractive index (RI) sensing performance of the microfiber MZI, a series of sucrose aqueous solution test systems with refractive index (1.3343 to 1.3423) were configured in this work. The spectral responses data were recorded in real time using an OSA. Initially, the sensing region of the MZI was immersed in a sucrose solution of specific concentration, and after spectral stabilization, an arbitrary interference peak was selected as the monitoring target to track its wavelength shift and intensity variation. After each test, the sensor was taken out and immersed in anhydrous ethanol solution for cleaning, so as to completely remove the residual sucrose solution on the surface and ensure the accuracy of subsequent measurement. By sequentially replacing sucrose solutions with different refractive indexes and repeating the above procedures, the response curve of the characteristic interference peak shift with the variation of ambient refractive index was obtained (**Figure S1a**). The experimental results showed a notable red shift in the interference wavelength, which migrated from approximately 1584 nm to 1602 nm as the ambient RI increased from 1.3343 to 1.3423. This phenomenon can be attributed to the theoretical mechanism wherein an increased ambient RI enhances the effective refractive index of the evanescent field on the microfiber surface, thereby altering the phase difference between the fundamental mode and the cladding mode, which ultimately induces a wavelength shift of the interference peak. Furthermore, the corresponding RI sensitivity was calculated by linear fitting, revealing a highly linear relationship between the wavelength shift and the ambient RI within the investigated range (Figure S1b). The refractive index sensitivity of the MZI is about 2307 nm/RIU. The results verified that the microfiber MZI has high sensitivity and excellent linear response characteristics in refractive index sensing applications.

Figure S1. MZI devices in different liquid environments (a) transmission spectrum and (b) linear fitting curve between wavelength shift and refractive index.

# 4. The stability of MZI devices


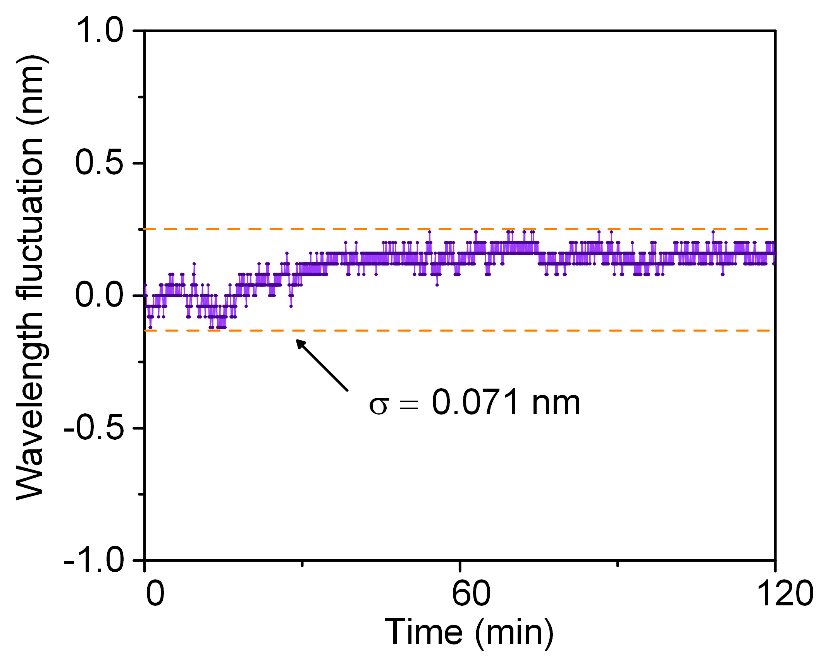


Figure S2. Peak wavelength fluctuation of the transmission spectrum of the proposed MZI interferometer is continuously irradiated for 120 min at the maximum power (586 mW/cm^2^) of 405 nm laser. The standard deviation measured over 120 min is 71 pm.

# 5. The process of PDMS encapsulation of microfiber sensors

PDMS is employed as a dielectric isolation layer owing to its simple synthesis, low cost, excellent thermal conductivity, optical transparency, flexibility, and biocompatibility, which collectively enable effective protection of components against environmental erosion and mechanical impact over a broad temperature range (–50 to 200 °C).^[^[^1^](#_ENREF_1)^]^ In addition, PDMS possesses a high thermo-optic coefficient,^[^[^2-3^](#_ENREF_2)^]^ meaning its refractive index is strongly influenced by temperature variations, an effect that can be accurately detected by the temperature-sensitive microfiber MZI. Therefore, in this work, the PDMS-encapsulated optical MZI was implemented to effectively isolate interference from ambient refractive index fluctuations while preserving high sensitivity to environmental temperature, thereby enabling precise monitoring of thermal effects in the material.

The PDMS used consists of pre-polymer A and a curing agent B. According to literature reports,^[^[^4-5^](#_ENREF_4)^]^ the mixing ratio of A and B significantly influences the thermal curing behavior of PDMS. To ensure good mechanical properties and thermal conductivity after curing for encapsulation purposes, the A/B components were mixed at a mass ratio of 10:1. Firstly, the mixture was stirred thoroughly until numerous tiny bubbles were generated. The mixture was then defoamed either by standing or centrifugation to ensure homogeneity and optical quality of the PDMS. Subsequently, the defoamed PDMS was dropped onto a clean glass substrate, scraped evenly and stood flat to form a thin film with uniform thickness as a packaging substrate. Finally, the prepared microfiber interferometer was fixed on the three-dimensional displacement platform fixture, and the position is adjusted to keep it straight. The sensing region of the interferometer was slowly immersed into the uncured PDMS, and both ends of the optical fiber were anchored onto the substrate to prevent deformation or fracture during encapsulation. The assembled structure was cured in an oven at 80 °C for 1 hour. A successfully PDMS-encapsulated microfiber is shown in **Figure S3**, exhibiting a smooth and uniform surface of the cured layer, which ensures mechanical stability and optical performance of the sensor.


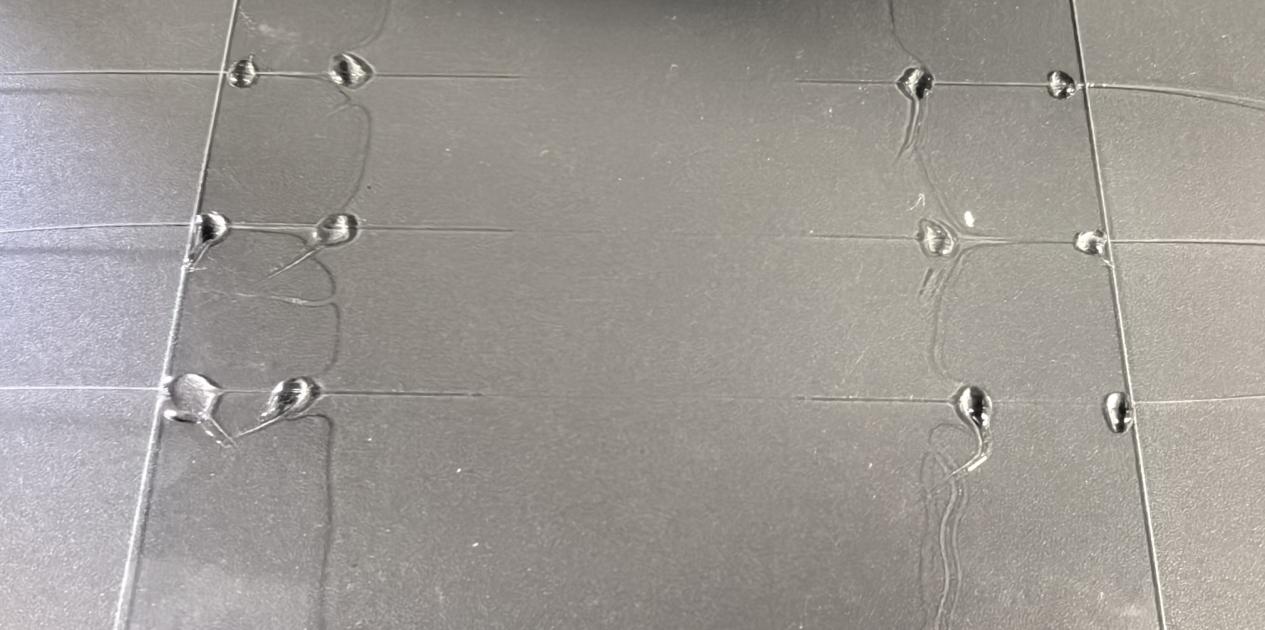


Figure S3. Schematic diagram of PDMS-encapsulated microfiber MZI.

# 6. Performance evaluation of encapsulated microfiber sensors

The temperature sensing performance of the encapsulated microfibers was measured using a high-precision small electronic temperature box. A series of temperature gradients were established at 5 °C intervals within the range of 26.5 to 60 ℃. After each temperature point remains stable, the interference spectrum was recorded via an OSA, and the wavelength shift of the interference peak was extracted for further analysis. As illustrated in **Figure S4a**, a noticeable blue shift in the interference spectrum was observed with increasing temperature. In this temperature range, the total wavelength shift of the interference peak reached 120 nm, showing obvious temperature dependence. This is attributed to the thermo-optic effect and thermal expansion coefficient of PDMS: the increase of temperature leads to the decrease of the refractive index of PDMS, and its thermal expansion leads to the change of the stress distribution of the microfiber, which causes the blue shift of the interference spectrum. As shown in Figure S4b (red line), the wavelength shift of the interference peak at different temperature exhibits a good linear relationship. The fitting equation is Y = −3.4935X + 88.444 (Y: wavelength shift/nm, X: temperature/℃), R² = 0.9914, temperature sensitivity is 3.4935 nm/℃. Furthermore, we further evaluated the response characteristics of the PDMS-encapsulated optic interferometer to the ambient refractive index. Within the refractive index range of 1.333 to 1.343, no significant wavelength shift was observed (Figure S4b, purple line), confirming that the PDMS encapsulation effectively isolates the sensing structure from external refractive index, consistent with the theoretical predictions. These results indicated that the PDMS packaging not only preserves high sensitivity to temperature but also effectively suppresses interference from ambient refractive index variations, thereby providing a viable platform for accurate temperature measurements in complex environments.

Figure S4. (a) The MZI spectrum of encapsulated microfiber changes with temperature. (b) The encapsulated microfiber MZI temperature sensitivity (red) and refractive index sensitivity (purple).

# 7. Preparation of Laser-Induced graphene

In this work, a continuous laser at a wavelength of 532 nm was used for the graphitizing modulation of commercial PI films. Firstly, the PDMS-encapsulated microfiber was horizontally positioned on a work stage, and an appropriate amount of PI solution was dispensed onto the PDMS surface using a rubber dropper. The device was placed in a drying oven at 80 °C for 1 hour to obtain a PDMS-encapsulated microfiber with a uniform surface covering a PI film. Then, a rectangular area of 1×2 cm, fully encompassing the sensing region of the microﬁber interferometer, was delineated on the PI film. The laser beam was then focused and aligned on the surface of this area. A computer-controlled platform and program were initiated to guide the laser spot in a bidirectional scanning motion along a predetermined path, starting from one corner of the rectangular area. Through this laser scanning process, localized graphitization of the PI film was achieved, ultimately forming a 1×2 cm rectangular region of LIG. After the laser scanning is completed, the non-graphitized PI surrounding the LIG area was carefully removed, leaving only the black rectangular LIG region intact. This LIG sample was reserved for use as the working electrode in subsequent photoelectrochemical reactions.

# 8. Performance Characterization of LIG

The structural characteristics of LIG were systematically investigated in detail by means of Raman spectroscopy. The Raman spectrum of the LIG (**Figure S5**) reveals three typical characteristic peaks: a D band (defect-induced peak) observed at approximately 1350 cm⁻^1^, a G band (vibration of sp²-hybridized carbon atoms in graphene) at around 1580 cm⁻^1^, and a 2D band (characteristic peak of graphene interlayer coupling) detected near 2700 cm⁻^1^. These characteristic peaks are highly consistent with previously reported LIG Raman spectroscopy results,^[^[^6-8^](#_ENREF_6)^]^ confirming the successful formation of graphene via laser-induced graphitization.





Figure S5. Raman spectrum of LIG.

Furthermore, the electrochemical performance of LIG was evaluated by cyclic voltammetry (CV) and electrochemical impedance spectroscopy (EIS). Compared with KCl solution without [Fe(CN)₆]³⁻/⁴⁻ (**Figure S6c**), typical oxidation and reduction peaks appeared in 0.5 M KCl solution containing 5 mM [Fe(CN)₆]³⁻/⁴⁻, corresponding to the redox reaction of [Fe(CN)₆]³⁻/[Fe(CN)₆]⁴⁻ (Figure S6a). Notably, the oxidation peak potential shifted positively while the reduction peak potential shifted negatively with increasing scan rates, a behavior primarily attributed to the diffusion-controlled kinetics of the redox species. Moreover, both the anodic and cathodic peak currents exhibited a linear relationship with the square root of the scan rate (Figure S6b), confirming fast electron transfer kinetics of the prepared LIG electrode.^[^[^9^](#_ENREF_9)^]^ Consistently, the EIS Nyquist plot (Figure S6d) revealed a lower interfacial charge transfer resistance for LIG, thereby indicating its superior charge transport capability.


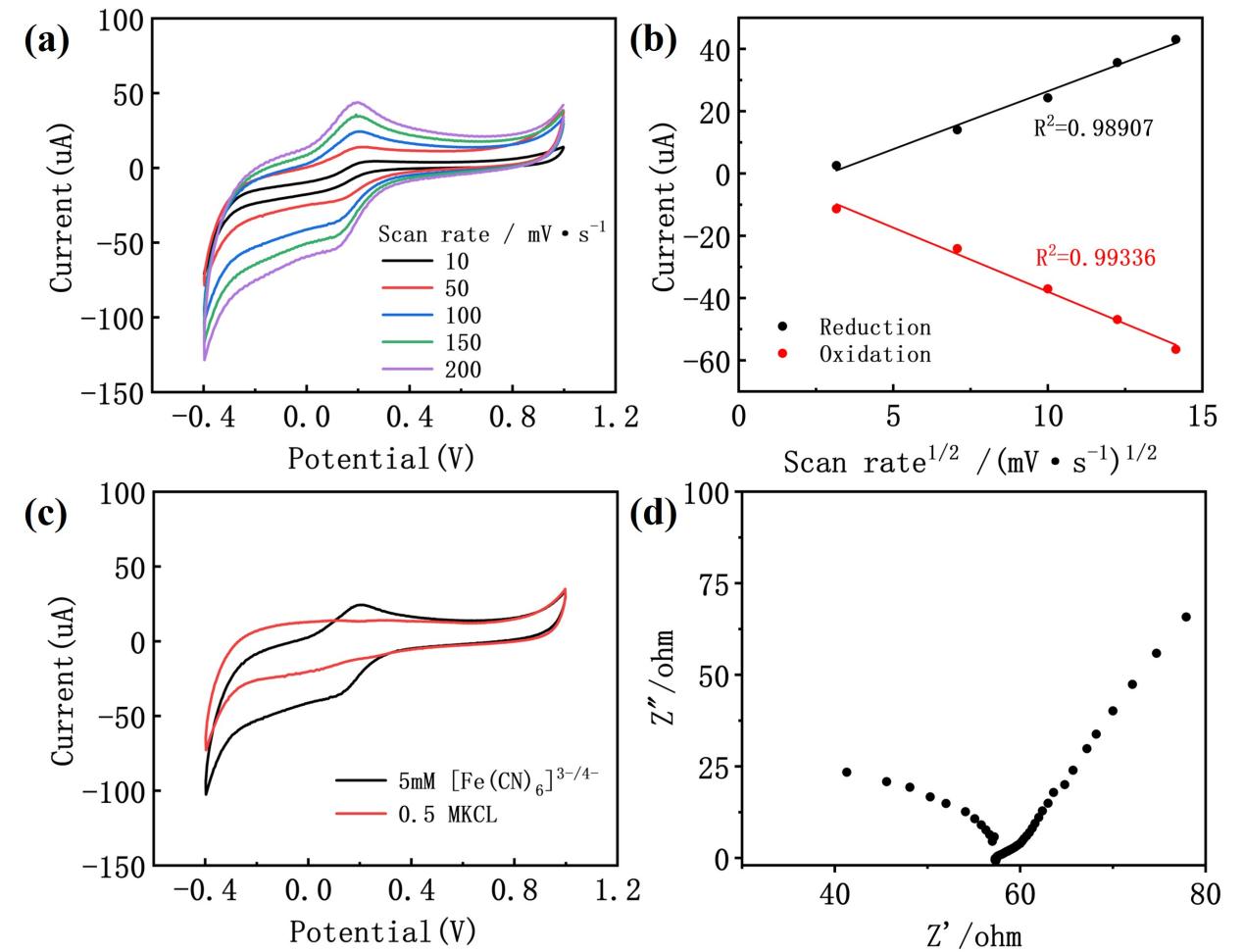


**Figure S6**. (a) Cyclic voltammetry curves of LIG as working electrode in 0.5 M KCl solution containing [Fe(CN)_6_]^3-/4-^ at different scan rates; (b) The square root relationship between the reduction oxidation peak current of LIG and the scanning rate; (c) CV comparison of 0.5 M KCl solution with or without [Fe(CN)_6_]^3-/4-^ at 50 mV/s scanning rate; (d) Impedance diagram of LIG.

We discussed the source of dark current through electrochemical tests, as shown in **Figure S7a**. CV curves of the LIG electrode in 0.2 M Na₂SO₄ electrolyte under dark conditions revealed no distinct redox peaks within the potential window from –0.2 V to 1.1 V (vs. SCE), which covers the operating potentials employed in this study. The CV profiles exhibited a nearly rectangular shape in the range of 0.25 V to 1.1 V (vs. SCE), characteristic of electric double-layer capacitive behavior and indicative of non-Faradaic processes.^[^[^10^](#_ENREF_10)^]^ In contrast, a weak and broad feature observed between 0.25 V and –0.3 V (vs. SCE) suggests the presence of sluggish Faradaic activity. As the scan rate increases, the current response is significantly enhanced, which is a typical characteristic of capacitive behavior (including double-layer capacitance and pseudocapacitance). The shape of the CV curve does not change much with the scan rate, maintaining a similar "hump" characteristic, indicating that the reaction process is a surface-controlled capacitive behavior. To clarify its nature, we compared CV curves of LIG in O₂ atmosphere and N₂-saturated electrolytes (Figure S7b). A lightly decrease in current density was observed in the O₂-free environment, indicating that the oxygen reduction reaction constitutes a component of the dark current. Furthermore, study has shown that the cyclic voltammetric response of carbon electrodes (such as glassy carbon and graphene) is related to the capacitive background and surface reactivity.^[^[^11^](#_ENREF_11)^]^ The abundant oxygen-containing functional groups ^[^[^12^](#_ENREF_12)^]^ and defective structures on the LIG surface can lead to charge localization and enhance intrinsic electrochemical activity,^[^[^13^](#_ENREF_13)^]^ potentially inducing additional surface-mediated redox reactions.^[^[^14^](#_ENREF_14)^]^ Therefore, the dark current in this system primarily originates from Faradaic processes associated with surface functional groups on LIG and the reduction of dissolved.


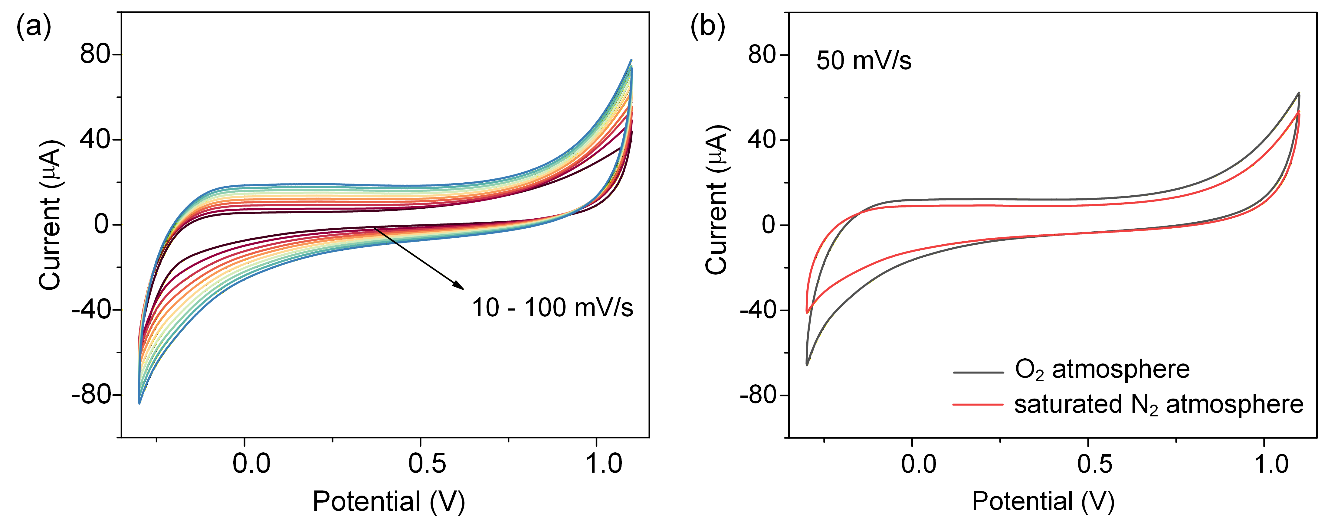


Figure S7. (a) CV curves of LIG in 0.2 M Na_2_SO_4_ electrolyte at different scan rates; (b) CV curves of LIG in Na_2_SO_4_ electrolyte under saturated N_2_ atmosphere and O_2_ atmosphere, scan rate 50 mV/s.

# 9. Photocurrent comparison between PDMS-MZI device and PDMS-MZI-LIG


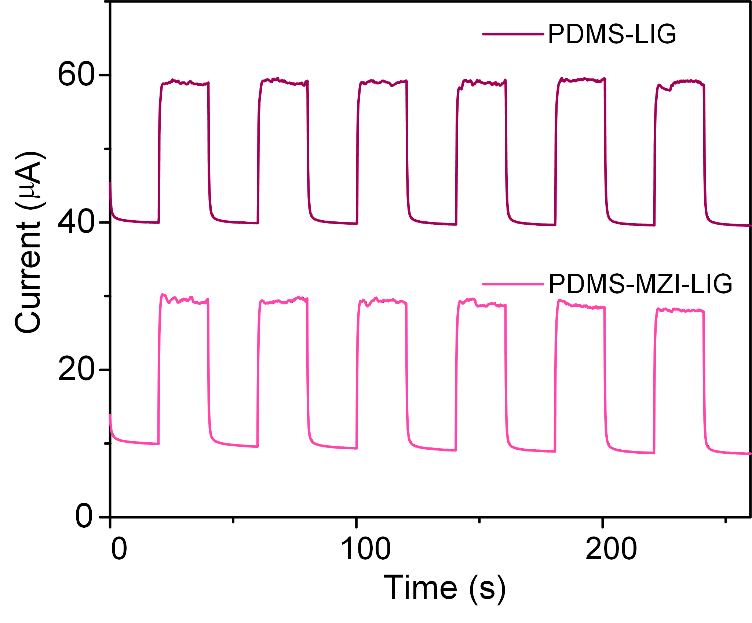


Figure S8. Photocurrent response of LIG electrode in 0.2 M Na_2_SO_4_ electrolyte with and without optical fiber.

# 10. Conversion of temperature variation into current variation


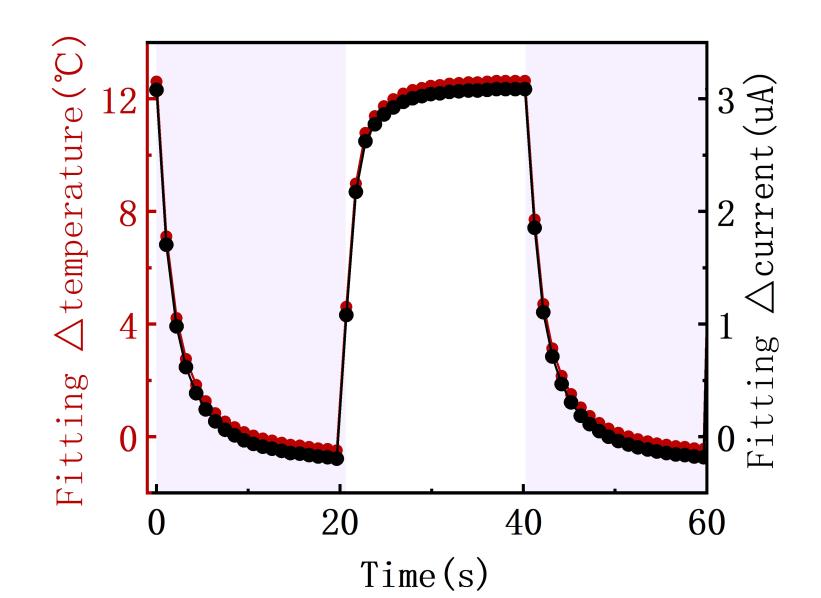


Figure S9. Temperature variation converted into current variation based on the fitted current-temperature relationship.

# 11. In-situ microfiber detection of photothermal effect of Ag and Ag/CuO electrode

We further performed a decoupling analysis of the photothermal and photoelectronic effects, based on our proposed in-situ decoupling strategy for photothermal effects during photoelectrochemical processes, for both the Ag electrode and Ag/CuO electrodes, respectively. We replaced the LIG electrode by drop-casting a layer of silver paste onto a PDMS film as an Ag electrode. Since the metal oxide CuO itself has poor conductivity and PDMS is insulating, to obtain a better electrochemical signal, we further drop-cast a CuO dispersion on the silver paste layer as an Ag/CuO electrode. Under the experimental conditions consistent with the manuscript, simultaneous electrochemical i-t measurements and spectral recordings were performed for the Ag and Ag/CuO electrodes at a laser power density of 339 mW/cm² and a bias of 0 V. The corresponding optical and electrical results are presented in **Figure S10**. As shown in Figure S10a and 10c**,** when the laser is turned on, the spectra of both Ag and Ag/CuO immediately show a significant blue shift, which tends to stabilize over time; then when the laser is turned off, the spectra immediately show a red shift, and the wavelength returns to the initial position. These spectral shifts are attributed to localized temperature changes at the electrode surfaces during the photoelectrochemical reaction, which aligns consistently with the findings of this study. The corresponding photocurrent variations from i-t measurements and wavelength shifts are shown in Figure S10b and S10d, respectively. For one on-off cycle, the wavelength shift induced by local thermal changes is approximately 30 nm (Ag electrode) and 40 nm (Ag/CuO electrode), indicating that the photothermal effect of the Ag/CuO electrode is improved compared to the Ag electrode. Furthermore, based on the temperature change obtained from the wavelength change, we quantitatively decoupled the proportions of the fast response current (RRC) and slow response current (SRC) of the Ag/CuO electrode in the total photocurrent at different laser power levels **(Figure S11**). As the laser power increased, the SRC ratio of Ag/CuO electrodes exhibited a marked increasing trend. This enhancement can be ascribed to the rising temperature change resulting from improved photothermal conversion at higher laser power. The increased temperature is conducive to increasing the number of activated molecules with high kinetic, rotational, and vibrational energies, thereby accelerating carrier generation and improving charge transfer efficiency, ultimately leading to a significant increase in photothermal current. These experimental results demonstrate that our optical sensor can also successfully monitor the surface temperature changes of these materials in situ and in real time during photoelectrochemical reactions, effectively decoupling their photothermal and photoelectric contributions. This further showcases the applicability of this technique for analyzing the dynamic thermal effects of different optoelectronic materials during operation.

*
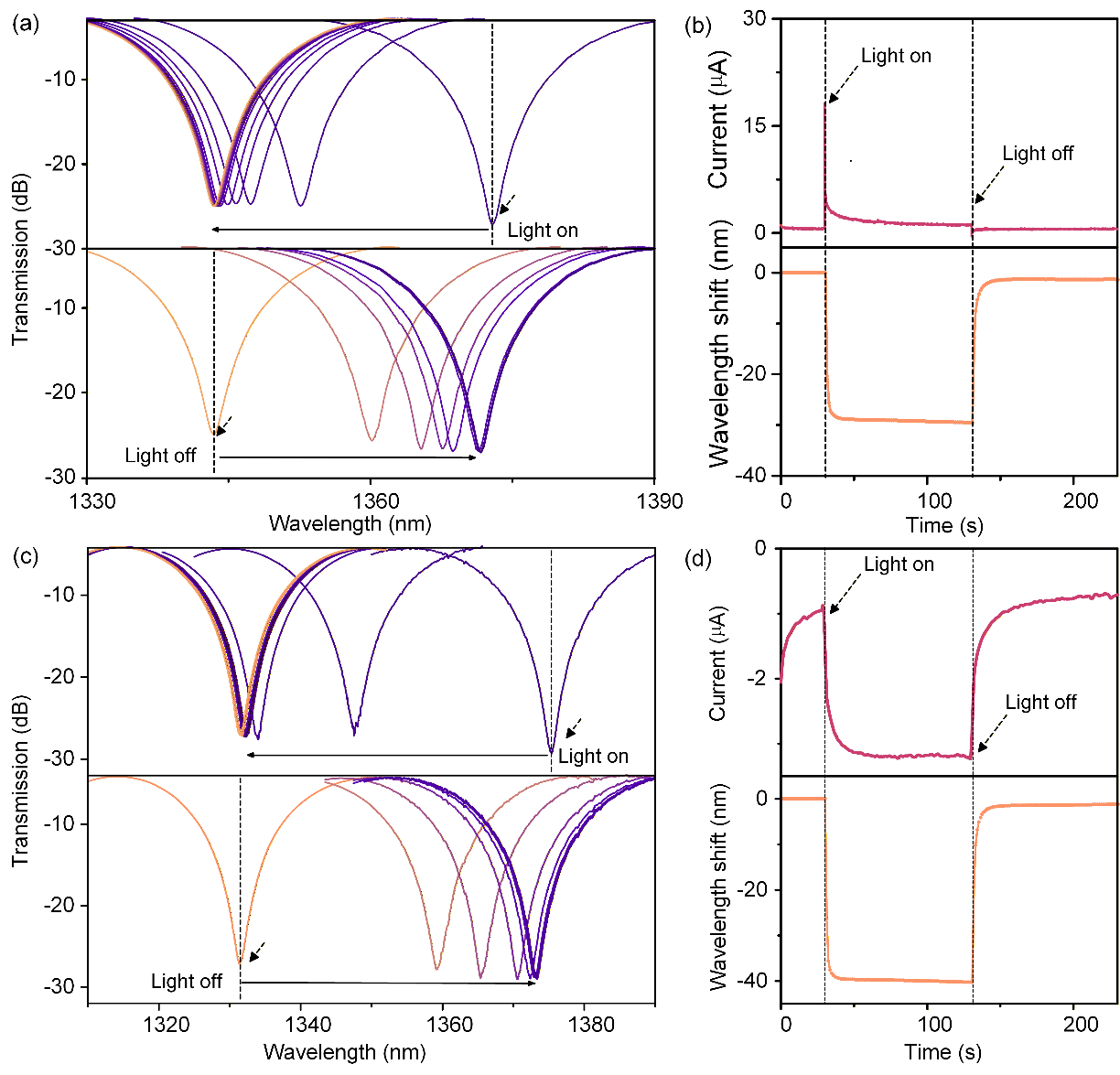
*

Figure S10. (a) Spectral shifts and (b) simultaneously recorded current response and wavelength variation of the Ag electrode under a laser power density of 339 mW/cm²; (c) spectral shifts and (d) corresponding current response and wavelength variation of the Ag/CuO electrode under identical illumination conditions (339 mW/cm²).

*
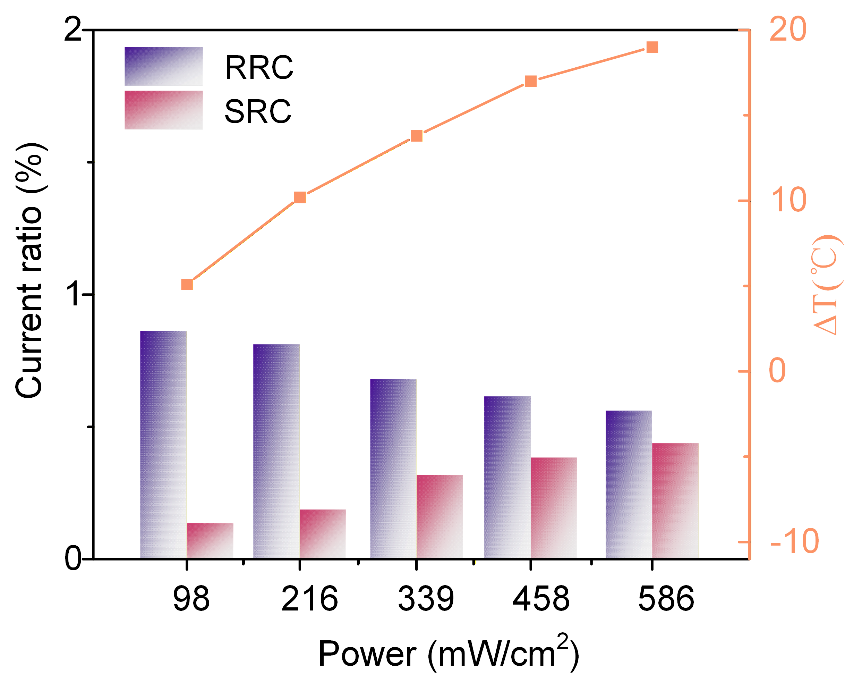
*

Figure S11. The proportion of the rapid response current (RRC) and slow response current (SRC) in the total photocurrent generated by Ag/CuO electrode under different laser power.

References

[1] F. Schneider, J. Draheim, R. Kamberger, U. Wallrabe, Process and Material Properties of Polydimethylsiloxane (PDMS) for Optical Mems, *Sens. Actuators, A* **2009**, *151*, 95.

[2] R.-j. Tong, Y. Zhao, H.-f. Hu, J.-f. Qu, Large Measurement Range and High Sensitivity Temperature Sensor with FBG Cascaded Mach-Zehnder Interferometer, *Opt. Laser Technol.* **2020**, *125*, 106034.

[3] D. Yi, F. Liu, Y. Geng, X. Li, X. Hong, High-Sensitivity and Large-Range Fiber Optic Temperature Sensor Based on Pdms-Coated Mach-Zehnder Interferometer Combined with Fbg, *Opt. Express* **2021**, *29*, 18624.

[4] A. Santiago-Alvarado, A. S. Cruz-Félix, J. González-García, O. Sánchez-López, A. J. Mendoza-Jasso, I. Hernández-Castillo, Polynomial Fitting Techniques Applied to Opto-Mechanical Properties of Pdms Sylgard 184 for Given Curing Parameters, *Mater. Res. Express* **2020**, *7*, 045301.

[5] A. Mata, A. J. Fleischman, S. Roy, Characterization of Polydimethylsiloxane (PDMS) Properties for Biomedical Micro/Nanosystems, *Biomed. Microdevices* **2005**, *7*, 281.

[6] W. Yang, M. Han, F. Liu, D. Wang, Y. Gao, G. Wang, X. Ding, S. Luo, Structure-Foldable and Performance-Tailorable Pi Paper-Based Triboelectric Nanogenerators Processed and Controlled by Laser-Induced Graphene, *Adv. Sci.* **2024**, *11*, 2310017.

[7] R. Xu, H. Lu, Z. Zheng, T. Zhou, In Situ Laser Direct Writing of Graphene-Based Layered Hybrid Materials with Superhydrophilicity, *ACS Appl. Mater. Interfaces* **2025**, *17*, 2436.

[8] P. Zhao, G. Bhattacharya, S. J. Fishlock, J. G. M. Guy, A. Kumar, C. Tsonos, Z. Yu, S. Raj, J. A. McLaughlin, J. Luo, N. Soin, Replacing the Metal Electrodes in Triboelectric Nanogenerators: High-Performance Laser-Induced Graphene Electrodes, *Nano Energy* **2020**, *75*, 104958.

[9] G. V. Govindaraju, G. P. Wheeler, D. Lee, K.-S. Choi, Methods for Electrochemical Synthesis and Photoelectrochemical Characterization for Photoelectrodes, *Chem. Mater.* **2017**, *29*, 355.

[10] A. M. Abdullah, M. A. S. Biswas, A. Dutta, J. Li, S. Das, X. Zhang, W. Zhang, F. T. Zohra, A. Moreno Calva, J. L. Gray, H. Jabelli, C. Wu, H. Cheng, In Situ Functionalized Mxene on Porous Laser-Induced Graphene for Adsorption-Dominated Miniaturized Multifunctional Sensors, *ACS Nano* **2025**, *19*, 33841.

[11] R. L. McCreery, Advanced Carbon Electrode Materials for Molecular Electrochemistry, *Chem. Rev.* **2008**, *108*, 2646.

[12] S. Liu, S. Lu, X. Zheng, X. Li, Z. Chen, L. Zhao, F. Xu, J. Shi, Laser-Induced Graphene/ITO Heterostructure for Self-Powered Ion Sensing Via Ionovoltaic Effect, *Chem. Eng. J.* **2025**, *525*, 170252.

[13] Z. Bie, Z. Jiao, X. Cai, Z. Wang, X. Zhang, Y. Li, W. Song, Pomegranate-Inspired Cathodes Mitigate the Mismatch between Carrier Transport and High Loading for Aqueous Zinc-Ion Batteries, *Adv. Energy Mater.* **2024**, *14*, 2401002.

[14] J. Li, C. Liu, X. Hu, J. Cai, H. Lian, D. Li, B. Zhong, W. Deng, H. Hou, G. Zou, X. Ji, Local Oxygen Reconstruction Enables Dual-Ion Active Sites in Carbon Cathode for High Energy Density Sodium-Ion Capacitors, *Adv. Funct. Mater.* **2025**, *35*, 2417059.
